# Deep Learning Analysis of Crystallization Using Polarized Light Microscopy and U‑Net Segmentation

**DOI:** 10.1021/acs.jpcb.5c03681

**Published:** 2025-09-17

**Authors:** Natalia Osiecka-Drewniak, Zbigniew Galewski, Marcin Piwowarczyk, Ewa Juszyńska-Gałązka

**Affiliations:** 1 Institute of Nuclear Physics, Polish Academy of Sciences, PL-31342 Kraków, Poland; 2 Faculty of Chemistry, University of Wrocław, PL-50383 Wrocław, Poland; 3 Research Center for Thermal and Entropic Science, Graduate School of Science, Osaka University, Toyonaka, Osaka 560-0043, Japan

## Abstract

Understanding the crystallization behavior of materials is essential to controlling their physical properties. In this study, we present an approach that combines polarized light microscopy with deep learning techniques to investigate the crystallization process of liquid-crystalline compound 9BA4. A U-Net convolutional neural network was trained to perform semantic segmentation of microscopy textures, enabling automated identification of crystalline (Cr) and smectic (SmC) phases during nonisothermal cooling performed at multiple cooling rates. The model outputs probability maps, which are binarized to quantify the degree of crystallization over the temperature. The crystallization kinetics were further analyzed by fitting a sigmoidal function to the experimental data, and the inflection point of the fitted curve was used to identify the temperature of maximum crystallization. The data were then fitted to the Ozawa model. The proposed methodology demonstrates the effectiveness of combining traditional optical techniques with neural-network-based image analysis to extract quantitative insights from complex texture evolution during phase transitions.

## Graphical abstract

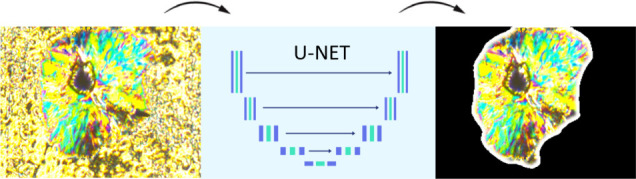

## 1 Introduction

Crystallization is a process that results in the formation of a solid phase characterized by long-range structural order. It represents a transition from a stochastic system dominated by thermal fluctuations to a deterministic system governed by thermodynamic principles.^1^ A phase transition is an abrupt change in a system that occurs over a small range in a control variable. For thermodynamic phase transitions, typical control variables include the temperature, pressure, and magnetic field. The crystallization process always arises from inseparable phenomena: nucleation and crystal growth, both governed by the balance between thermodynamic and kinetic factors.^2^ Nucleation serves as the starting point of crystallization, beginning with the formation of a nucleus composed of a small cluster of molecules. This nucleus then grows from microscopic to macroscopic dimensions.^3^ Each substance exhibits a specific temperature range in which optimal conditions for nucleation and subsequent crystal growth are achieved. Advancements in understanding crystallization behavior primarily stem from studies focused on the overall degree of crystallization, which reflect the kinetics of crystal growth.^4^

There are many experimental methods that allow the determination of the degree of crystallization *D*(*t*). One of them is the observation of textures under a polarizing microscope. The degree of crystallization is then determined by eq 1

1 $$D\left(T\right)=\frac{S_{Cr}\left(T\right)}{S}$$

where *S*_*Cr*_ is the area of the texture corresponding to the crystal phase during a change in temperature *T* and *S* is the total surface area of the texture.^5^ However, manually determining which area of the texture belongs to each phase can be time-consuming, especially when the textures are similar to each other. The aim of this article is to present a method using U-net architecture neural networks to automatically determine the degree of crystallization.

U-Net is a convolutional neural network architecture originally developed for biomedical image segmentation. The network has a U-shaped structure consisting of a contracting path encoder and an expansive path decoder. The contracting path is responsible for capturing context through repeated application of convolutional layers and maximum pooling, while the expansive path enables precise localization using up-sampling operations. A key feature of U-Net is the use of skip connections, which directly link the corresponding layers in the encoder and decoder paths. These connections help retain spatial information that may be lost during down-sampling.^6^ U-Net is particularly well-suited for tasks with limited training data, making it highly effective in domains such as medical imaging and microscopic image analysis. Its ability to perform pixel-wise classification makes it ideal for segmenting complex textures or structures.^7,8^ The U-Net architecture is widely used in the study of crystals and in materials engineering, particularly for tasks such as image segmentation, structural analysis, and defect detection.^9,10^ In this publication, we will show how the U-net neural network may be used to identify phase transitions and crystallization regions in polarizing microscopy images.

In this article, we are going to focus on 4-nonyloxybenzylidene-4′-propylaniline (9BA4). The molecule C_9_H_19_OC_6_H_4_­CHNC_6_H_4_C_4_H_9_ consists of one alkyloxy chain and one alkyl chain which are connected by an azomethine brige. During cooling, the 9BA4 nematic phase (383.2–379.8 K) and smectic C (SmC) phase (379.8–356.5 K) have been observed. At a temperature point of 360 K, the crystallization process starts (Figure 1).^11^

**Figure 1:**
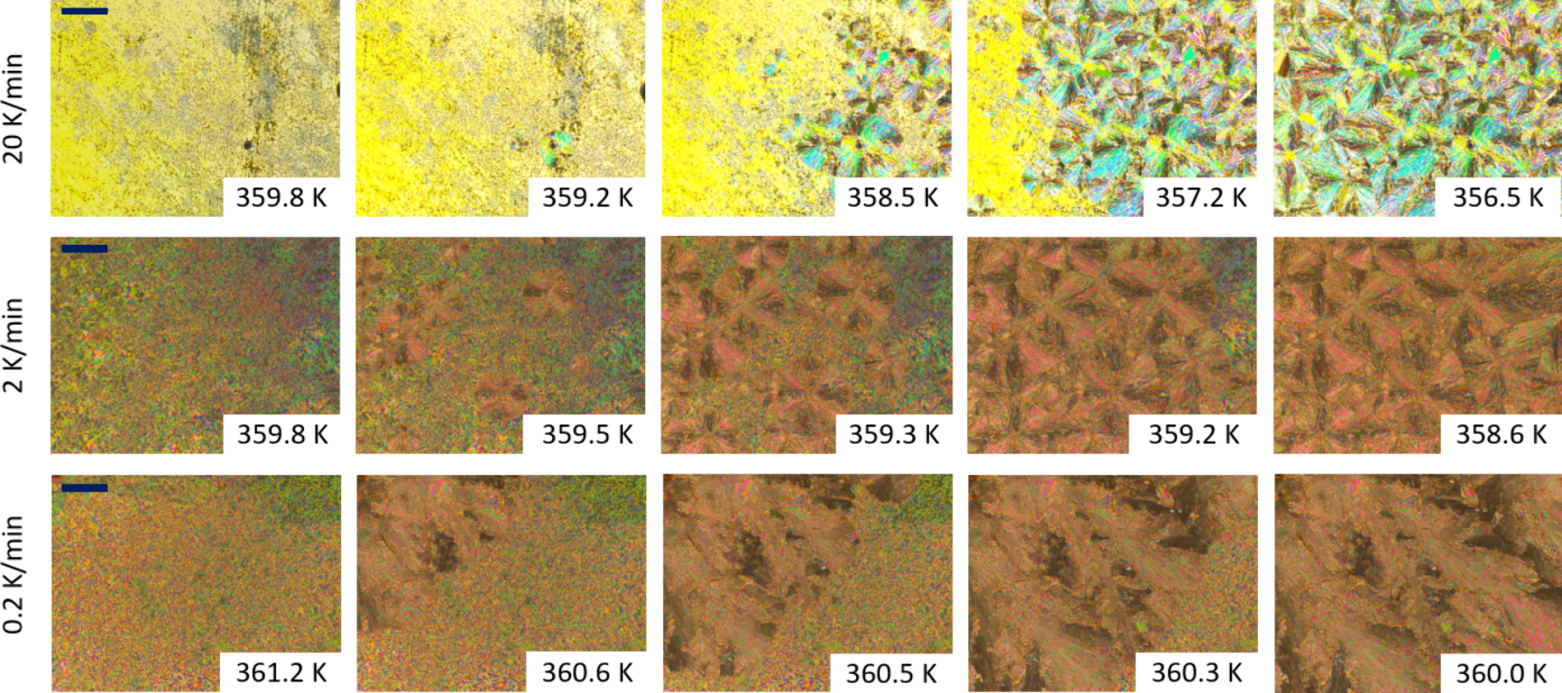
Textures of substance 9BA4 observed during the crystallization process for selected cooling rates and temperatures. On the far left are textures of the SmC phase, and on the far right are textures of the Cr phase. The length of the navy-blue bar in the upper left corner of the images on the left corresponds to 200 μm.

## 2 Materials and Methods

The textures were obtained through observations under a polarizing optical microscope (POM) of the 9BA4 sample. Substance 9BA4 was synthesized by Prof. Z. Galewski. POM observations were conducted by using a Leica DM2700P polarizing microscope. The temperature was controlled with a Linkam T96-S device. The samples were placed on glass slides and observed from 353 to 173 K with cooling rates of 20, 5, 2, 1, 0.5, 0.2, and 0.1 K/min.

The code used in this study was implemented in Python using the Tensorflow^12^ and OpenCv^13^ libraries. This article utilized the U-Net architecture, as thoroughly described in Table 1. Training was conducted for between 15 and 50 epochs, stopping once an accuracy of 98% was achieved, with a batch size of 20. Accuracy was used as the monitoring metric during training. The model’s hyperparameter optimization was conducted using the Google Colaboratory environment.^14^ The model was trained without the use of cross-validation or any form of regularization. Training was performed using the Adam optimizer (learning rate = 0.0001), with binary cross-entropy as the loss function. In the U-Net architecture, several key layers work together to perform effective image segmentation. The input layer receives the image data, typically in the form of a 2D array with one or more channels. The Conv2D (2D convolution) layers extract features such as edges, textures, and structures from the image by applying learnable filters. These layers are usually applied twice in succession within each block to capture more complex features. The MaxPooling2D layers reduce the spatial dimensions of the feature maps, effectively summarizing the most important information and allowing the network to focus on larger patterns. This down-sampling occurs in the contracting path of the U-Net. To restore the original resolution of the image, UpSampling2D layers are used in the expansive path, increasing the size of the feature maps. A crucial element of U-Net is the use of concatenated layers, which create skip connections by combining feature maps from the contracting path with those in the corresponding layers of the expansive path. This helps preserve spatial information that might otherwise be lost during down-sampling, improving the accuracy of the final segmentation.^15,16^

**Table 1:** Summary of the Used U-Net Neural Network^a^

Layer (type)	Output shape	Number of optimized parameters	Connected to
input_layer (Input Layer)	(None, 256, 256, 3)	0	-
conv2d (Conv2D)	(None, 256, 256, 64)	1792	input_layer
conv2d_1 (Conv2D)	(None, 256, 256, 64)	36928	conv2d
max_pooling2d (MaxPooling2D)	(None, 128, 128, 64)	0	conv2d_1
conv2d_2 (Conv2D)	(None, 128, 128, 128)	73856	max_pooling2d
conv2d_3 (Conv2D)	(None, 128, 128, 128)	147584	conv2d_2
max_pooling2d_1 (MaxPooling2D)	(None, 64, 64, 128)	0	conv2d_3
conv2d_4 (Conv2D)	(None, 64, 64, 256)	295168	max_pooling2d_1
conv2d_5 (Conv2D)	(None, 64, 64, 256)	590080	conv2d_4
max_pooling2d_2 (MaxPooling2D)	(None, 32, 32, 256)	0	conv2d_5
conv2d_6 (Conv2D)	(None, 32, 32, 512)	1180160	max_pooling2d_2
conv2d_7(Conv2D)	(None, 32, 32, 512)	2359808	conv2d_6
up_sampling2d (UpSampling2D)	(None, 64, 64, 512)	0	conv2d_7
concatenate (Concatenate)	(None, 64, 64, 768)	0	up_sampling2d
conv2d_8 (Conv2D)	(None, 64, 64, 256)	1769728	concatenate
conv2d_9 (Conv2D)	(None, 64, 64, 256)	590080	conv2d_8
up_sampling2d_1 (UpSampling2D)	(None, 128, 128, 256)	0	conv2d_9
concatenate_1 (Concatenate)	(None, 128, 128, 384)	0	up_sampling2d_1
conv2d_10 (Conv2D)	(None, 128, 128, 128)	442496	concatenate_1
conv2d_11 (Conv2D)	(None, 128, 128, 128)	147584	conv2d_10
up_sampling2d_2 (UpSampling2D)	(None, 256, 256, 128)	0	conv2d_11
concatenate_2 (Concatenate)	(None, 256, 256, 64)	0	up_sampling2d_2
conv2d_12 (Conv2D)	(None, 256, 256, 64)	110656	concatenate_2
conv2d_13 (Conv2D)	(None, 256, 256, 64)	36928	conv2d_12
conv2d_14 (Conv2D)	(None, 256, 256, 1)	65	conv2d_13

a The description of U-Net layers is provided in the Materials and Methods section.

## 3 Results and Discussion

To train a U-Net model, data preparation was performed separately for each cooling rate, starting with collecting paired input images and the corresponding segmentation masks, where each mask indicates the class of each pixel (e.g., background or object). Both images and masks should have the same dimensions. The next step is preprocessing, which includes resizing the images and masks to a consistent shape (e.g., 256 × 256), normalizing pixel values (e.g., scaling to [0,1]), and ensuring that masks are in the correct format (binary or categorical). Data augmentation is often applied to artificially expand the data set and improve model generalization; this involves operations like rotation, flipping, scaling, and brightness adjustments, applied identically to both images and masks. Finally, the data set is split into training, validation, and test sets—typically in a 70/15/15 ratio—to allow for effective training and evaluation of the model.^17^

To train a neural network, a data set containing several thousand examples is typically required.^18^ When training a network with the U-Net architecture, it is also necessary to provide the model with the corresponding mask information. Preparing such a data set for the purpose of studying the crystallization process would be very time-consuming, and the resulting model would likely be applicable only to the specific compound used for training or to other compounds with phases that exhibit similar textures under polarized light. For this reason, in this article, we propose an alternative approach. For each cooling rate, two images of the SmC phase were selected at temperatures approximately 2 and 4 K above the onset of the crystallization process, and two images of the Cr phase were selected at temperatures approximately 2 and 4 K below the completion of crystallization. In the next step, we concatenate textures of the SmC phase and Cr phase in such a way that the texture of SmC was a background, and on them we placed randomly the texture of the Cr phase in the shape of a circle or rectangle (Figure 2). The sizes of the circles and squares were selected randomly. The radius of the circles was chosen to be between 1 and 400 pixels, while the side length of the squares was randomly selected to be between 2 and 400 pixels. In this way, 20000 textures and their corresponding masks were generated. The data was divided into training, validation, and test sets in the following proportion: 80/10/10. The U-Net neural networks were trained until they reached at least 98% prediction accuracy (Figure 3). Figure 4 shows how the U-Net network classifies the texture of 9BA4 during the crystallization process. As a result, a matrix with values ranging from 0 to 1 is obtained, representing the probability of assigning each pixel to the Cr phase. This matrix must be binarized: values below 0.5 are assigned to 0 (SmC phase), while values above 0.5 are assigned to 1 (Cr phase). The outcome of this procedure is shown in Figure 5. It is interesting that although the neural network was trained on symmetrical shapes it was able to accurately reproduce the irregular shape of the crystallizing phase.

**Figure 2:**
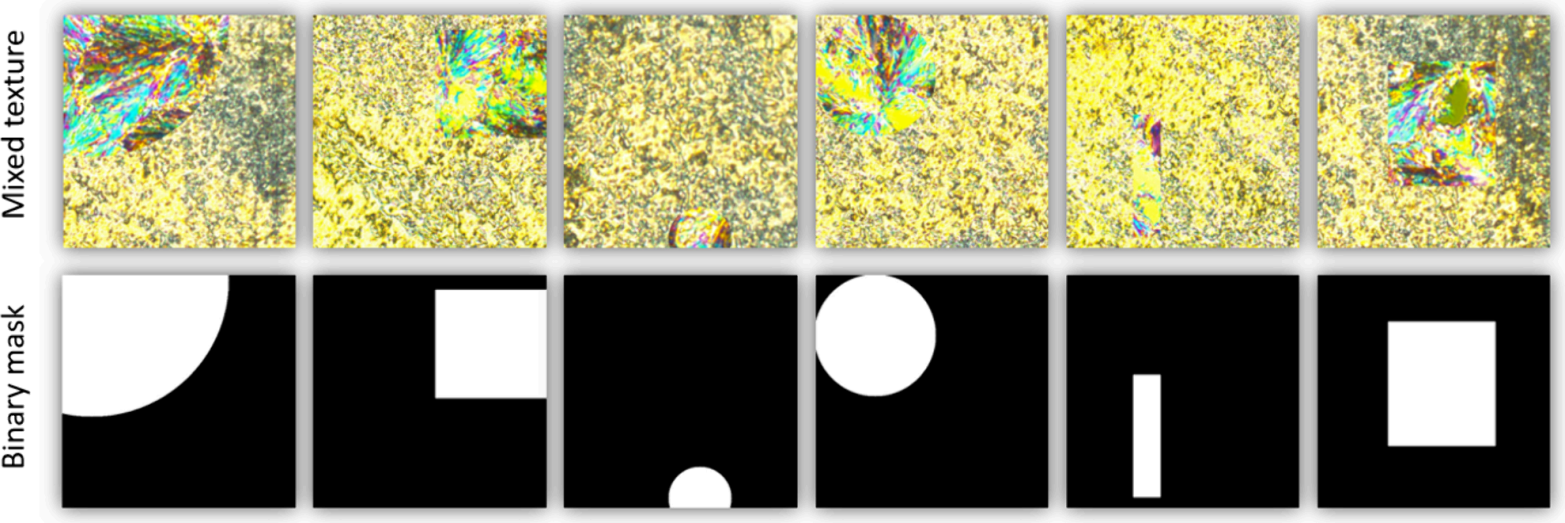
Example of concatenated textures (upper row) and the corresponding binary mask where white corresponds to the Cr phase and black refers to the SmC phase (bottom row). Observed under a cooling rate of 20 K/min.

**Figure 3:**
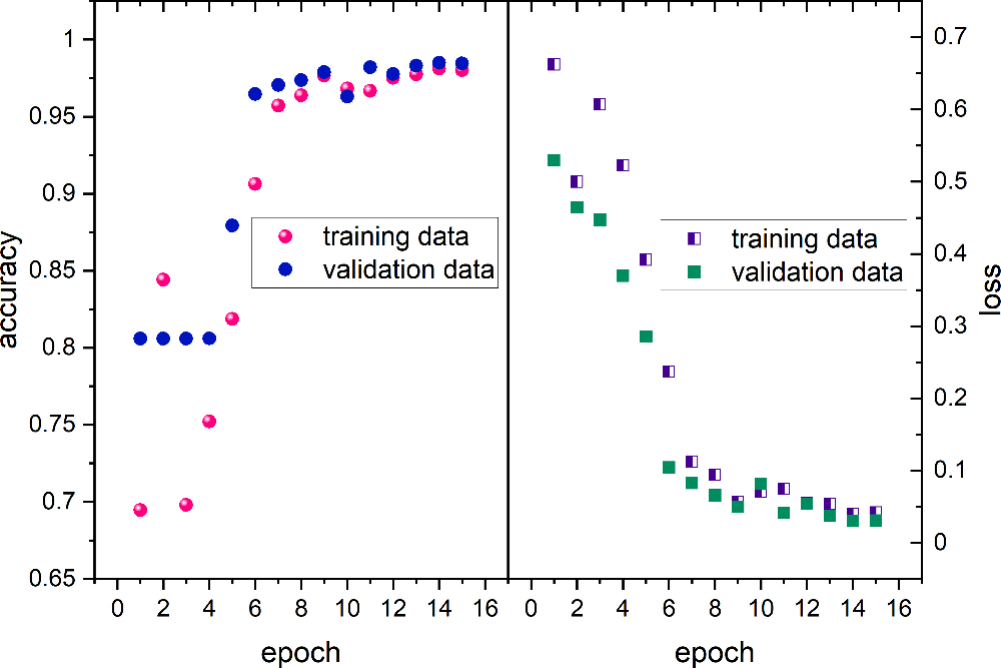
Accuracy and loss score of the U-Net neural network for training and validation data. Model reach the accuracy of 98.5%. The data were neither overfitted nor underfitted. Observed under a cooling rate of 20 K/min.

**Figure 4:**
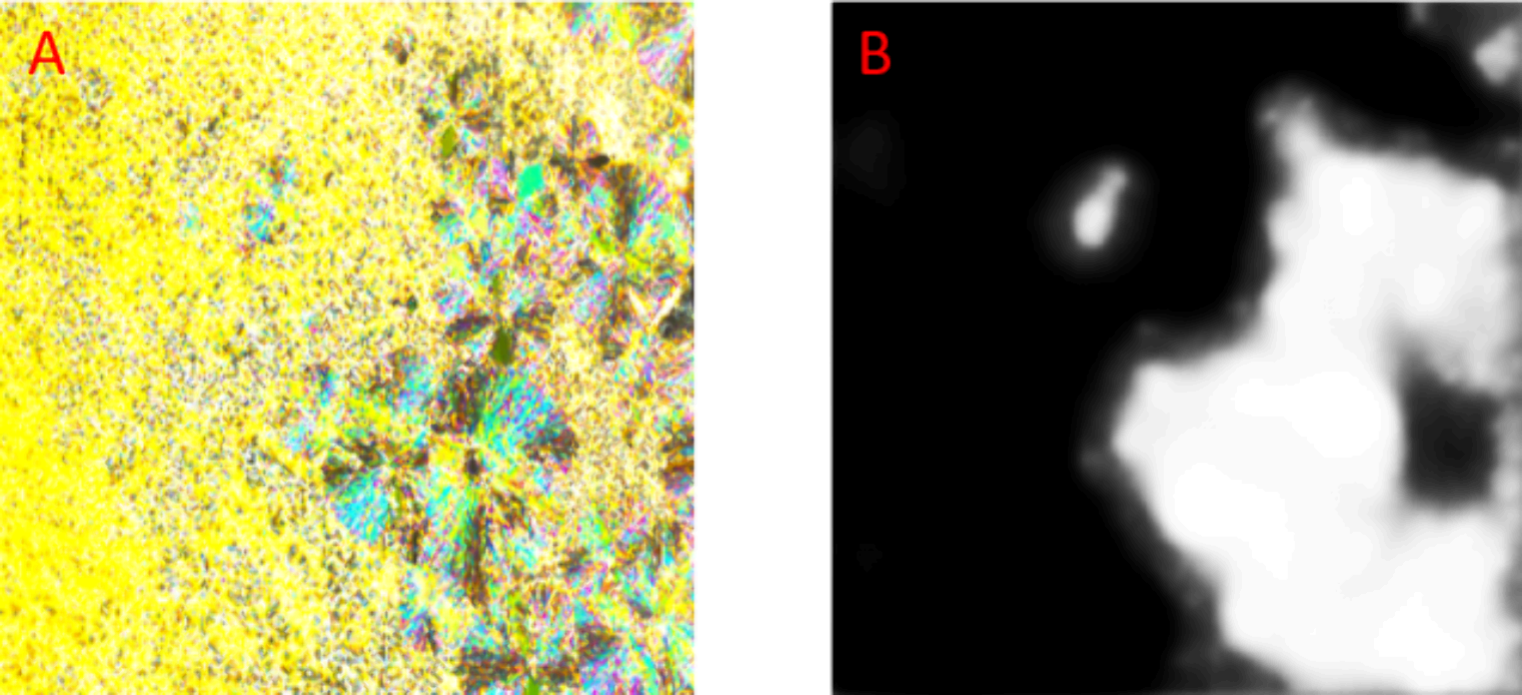
Comparison of original image observed at a temperature of 358.5 K under a cooling rate of 20 K/min (A) and predicted by the U-Net network mask (B). The diagonals of the presented images measure 1600 μm.

**Figure 5:**
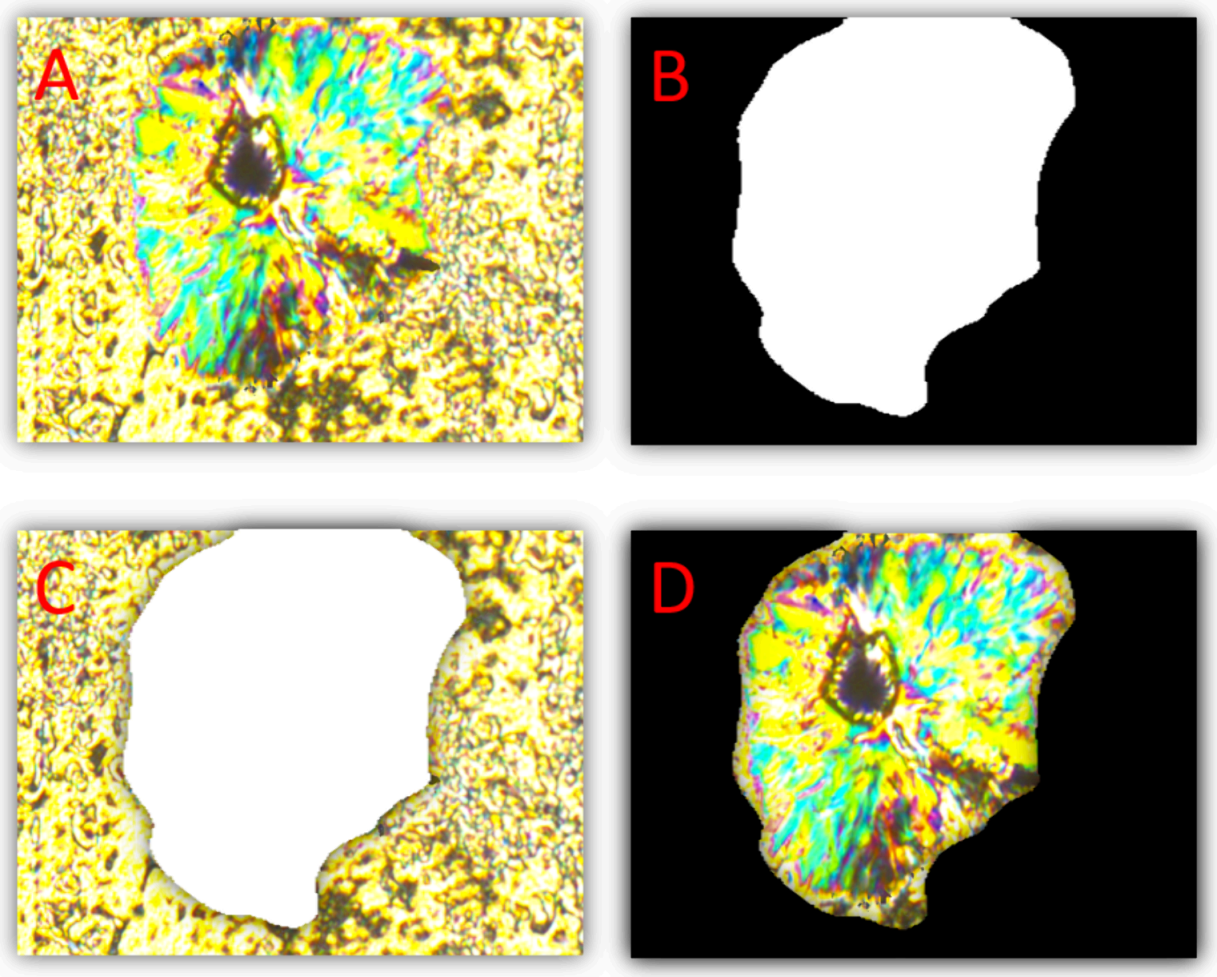
(A) Original texture of a binary system (SmC and Cr) observed at a temperature 359.2 K under a cooling rate of 20 K/min. (B) Result of the Unet prediction (black corresponds to SmC and white corresponds to the Cr phase). (C and D) Combinations of original texture and mask. The diagonals of the presented images measure 320 μm.

The degree of crystallization of 9BA4 was determined by modifying eq 1, where the numerator contains the sum of values from the binarized mask and the denominator is the product of the mask’s dimensions. The degree of crystallization obtained for several cooling rates is presented in Figure 6. The crystallization curve exhibits a sigmoidal (S-shaped) profile, which is characteristic of phase-transition processes.^19^ Initially, the degree of crystallinity increases slowly with decreasing temperature, followed by a rapid growth phase, where crystallization accelerates. This is then followed by a plateau as the material approaches complete crystallization. A sigmoidal function with the following form (eq 2) was fitted to the data

2 $$D\left(T\right)=\frac{a}{1+e^{\left[-k\left(T-T_{0}\right)\right]}}$$

where *D*(*T*) is the degree of crystallization at temperature *T*, *a* is the maximum degree of crystallization, *T*_0_ is the temperature at which *D*(*T*) = *a*/2, and *k* is the steepness coefficient of the curve. Calculating the derivative of the fitted sigmoidal function allowed us to determine the inflection point, which corresponds to the temperature at which the crystallization rate is at its maximum.^20^ An example of fitting the sigmoidal function and determining its first derivative is shown in Figure 7 for the sample cooled at a rate of 20 K/min.

**Figure 6:**
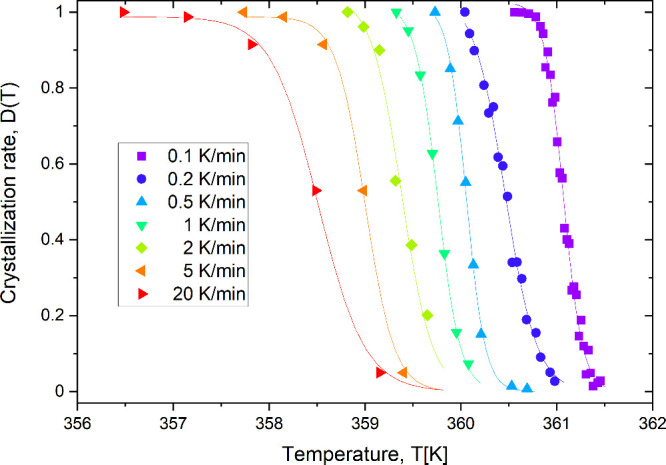
Degree of crystallization as a function of temperature for selected cooling rates of the 9BA4 sample.

**Figure 7:**
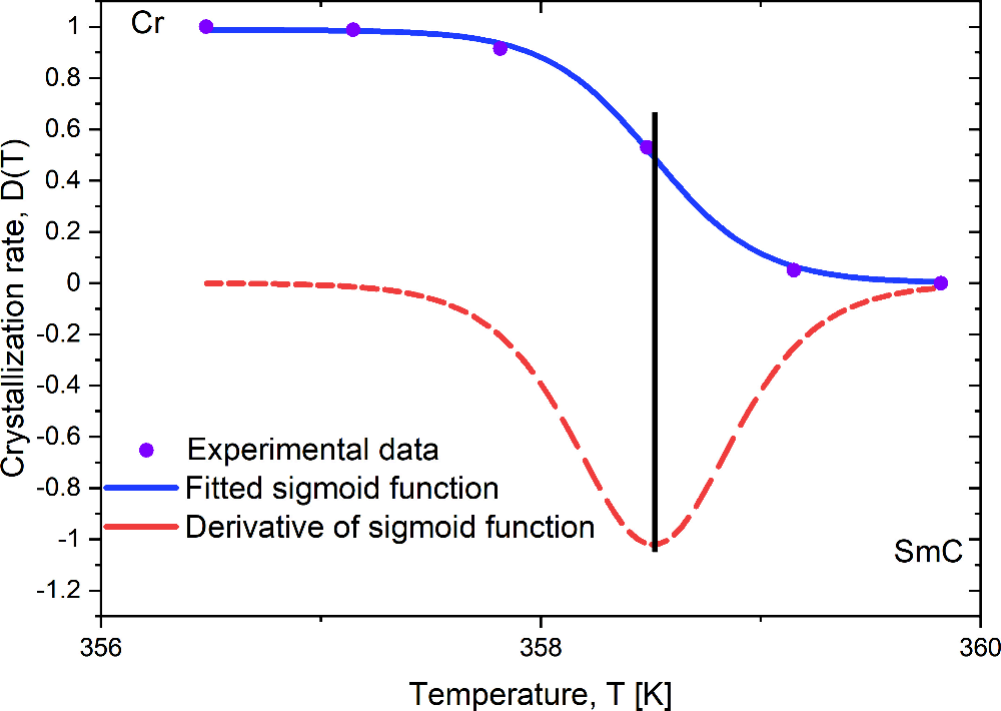
Crystallization rate of 9BA4 for 20 K/min cooling rate, sigmoid fit to crystallization rate, and derivative of sigmoid function.

For the examined compound, the fitted sigmoidal parameters (eq 2) reveal systematic trends with cooling rate in Table 2. The inflection temperature *T*_0_, corresponding to 50% crystallinity, shifts from 358.51 K at the fastest cooling rate (20 K min^–1^) to 361.07 K at the slowest rate (0.1 K min^–1^). This upward shift suggests that slower cooling allows crystallization to occur at higher temperatures, likely due to an extended time for nucleation and growth before the sample reaches lower thermal energy states. The steepness parameter *k*, which reflects how rapidly crystallization proceeds with temperature change, also varies with the cooling rate. Lower *k* values (e.g., 4.13 at 20 K min^–1^) indicate a broader temperature range for crystallization, whereas higher values (e.g., 10.09 at 0.1 K min^–1^) imply a narrower window and more abrupt transition through the crystallization regime. This behavior suggests that slower cooling promotes a more cooperative and synchronized crystallization process, consistent with well-defined nucleation and growth with minimal kinetic dispersion. At high cooling rates, crystallization is initiated later (lower *T*_0_) and proceeds more gradually, likely due to a reduced time for stable nuclei formation at higher temperatures. Conversely, at low cooling rates, the system approaches equilibrium more closely, enabling crystallization to occur within a tighter temperature range at higher *T*_0_. Overall, the observed trends are characteristic of a thermally activated process whose kinetics are strongly modulated by the rate of temperature change.

**Table 2:** Parameters of the Sigmoidal Function Fit and Ozawa Model to the Crystallization Degree Function for the Selected Sample Cooling Rates

Cooling rate, φ [K/min]	*k*	*T*_0_ [K]
20	4.13	358.51
5	6.62	359.00
2	6.31	359.38
1	7.24	359.75
0.5	8.47	360.06
0.2	6.07	360.46
0.1	10.09	361.07

The Ozawa model (eq 3) is a widely used approach to describe nonisothermal crystallization kinetics, extending the Avrami formalism to conditions where the temperature changes continuously during the process. In this model (eq 3), the relative degree of crystallinity *D*(*T*) at a given temperature is expressed as

3 $$1-D\left(T\right)=e^{-K\left(T\right)/\varphi^{m}}$$

where ϕ is the cooling rate, *m* is the Ozawa exponent related to the nucleation mechanism and dimensionality of crystal growth, and *K*(*T*) is a temperature-dependent rate constant. By taking the logarithm of both sides, the model enables linearization, making it possible to determine *m* and *K*(*T*) from experimental data collected at multiple cooling rates. The Ozawa model assumes that the overall crystallization process under nonisothermal conditions can be described by the superposition of instantaneous isothermal crystallization rates corresponding to each temperature. This approach is particularly useful for both polymeric and small-molecule systems, as it provides insight into the influence of the cooling rate on crystal growth kinetics and allows the identification of mechanisms dominating nucleation and growth under practical processing conditions. In the Ozawa model, the exponent *m* characterizes the dimensionality of crystal growth and the nature of the nucleation process under nonisothermal conditions. Conceptually, *m* is analogous to the Avrami exponent used in isothermal crystallization analysis, but it is determined from cooling experiments at different rates. Its value depends on both the geometry of crystal growth (one-, two-, or three-dimensional) and whether nucleation is instantaneous or occurs continuously during the process.^21,22^

The result of fitting the Ozawa model to the experimental data obtained from polarizing microscopy by using the U-Net architecture is shown in Figure 8. The Ozawa model is applied to measurements performed at the same temperature for different cooling rates. Therefore, to use this model, we selected the temperature range between 359 and 360 K, as this range contains crystallization degree curves obtained for cooling rates of 20, 5, 2, 1 and 0.5 K/min. Due to technical limitations of polarizing microscopy observations, where images can be recorded only every 3 s, the required data were taken from the sigmoidal fits to the crystallization curves described by eq 2. The logarithmic plots of the model fits are straight lines. For temperatures from 359.0 to 359.6 K, these lines are parallel to each other, as the same *m* value of 1.4 ± 0.2 was obtained for these data. At 359.8 K, this coefficient is 2.4 ± 0.2, and at 360 K, *m* equals 2.1 ± 0.2. The change in the *m* coefficient is reflected in the change in the slope of the lines relative to each other in Figure 8. The *m* value in the Ozawa model for the 9BA4 compound lies between 1 and 2 in the temperature range of 359.0–359.6 K and increases toward 2 with rising temperature above 359.8 K, which suggests a transition in the crystallization mechanism. At lower *m* values, the process likely involves a mixed growth geometry with limited nucleation or reduced dimensionality, resulting in weaker sensitivity to the cooling rate. As *m* approaches 2, the growth behavior becomes closer to two-dimensional, indicating more synchronized nucleation and growth and a stronger dependence on the cooling rate. This trend may reflect a kinetic transition where higher thermal energy promotes more complete structural development and reduces diffusion limitations.^3^

**Figure 8:**
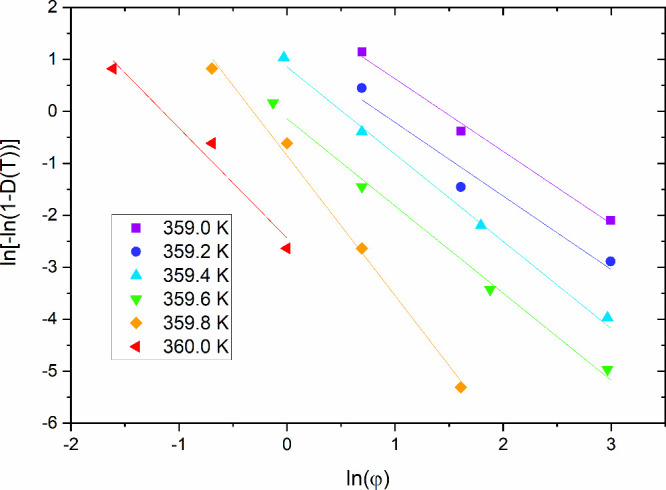
Result of fitting the experimental data of crystallization rate to the Ozawa model for the 9BA4 compound.

The U-Net architecture has not yet been used in the study of the crystallization process of soft mater compounds. U-Net has been utilized in the study of crystallization in metallic materials, particularly through the segmentation and analysis of microstructural images. While it is not typically used to directly compute the numerical degree of crystallinity (e.g., a value of between 0 and 1), U-Net enables accurate identification and classification of crystalline phases within metallographic or electron microscopy images. This allows researchers to estimate the volume fraction of crystalline regions, which corresponds to the degree of crystallization. Additionally, U-Net-based models have been applied to segment grain boundaries and analyze grain growth, nucleation density, and phase transformation kinetics. These analyses provide indirect but valuable insight into the crystallization process. For instance, enhanced U-Net architectures have been used to process SEM images, facilitating the extraction of grain orientation and distribution, which are key indicators of crystallographic ordering. Such applications are increasingly common in both fundamental research and industrial metallurgy, enabling an automated, high-precision assessment of crystallinity in complex metallic systems.^23,24^

## 4 Conclusions

The U-Net neural network effectively segments microscopy images even when trained on simplified symmetrical shapes. It can accurately recognize irregular crystalline phase boundaries, indicating strong generalization capabilities. The use of U-Net enables automated and rapid phase identification in polarized light microscopy images, eliminating the need for manual annotation and significantly accelerating data analysis. By binarizing the probability maps generated by the network, it was possible to quantitatively evaluate the degree of crystallization based on the proportion of pixels assigned to the crystalline (Cr) phase. The proposed approach can be easily extended to other liquid-crystalline compounds or material classes provided polarized microscopy images are available. In the future, the method could be further developed by training models in different phases. The model was applied for different cooling rates, which enabled the fitting of the Ozawa model. The *m* parameter from the Ozawa model was 1.4 for temperatures in the range 359.0–359.6 K and increased to 2.0 at 359.8 K and then to 2.2 at 360 K. This increase in *m* suggests a shift in the crystallization mechanism from mixed-growth geometry with limited nucleation at lower temperatures to more two-dimensional, synchronized growth at higher temperatures, indicating stronger sensitivity to the cooling rate and reduced diffusion limitations.
